# Integrating evolutionary, developmental and physiological mismatch

**DOI:** 10.1093/emph/eoad023

**Published:** 2023-08-05

**Authors:** Paul E Griffiths, Pierrick Bourrat

**Affiliations:** Department of Philosophy and Charles Perkins Centre, The University of Sydney, Sydney, Australia; Department of Philosophy, Macquarie University, North Ryde, Australia; Department of Philosophy and Charles Perkins Centre, The University of Sydney, Sydney, Australia

## Abstract

Contemporary evolutionary medicine has unified the idea of ‘evolutionary mismatch’, derived from the older idea of ‘adaptive lag’ in evolution, with ideas about the mismatch in development and physiology derived from the Developmental Origins of Health and Disease (DOHaD) paradigm. A number of publications in evolutionary medicine have tried to make this theoretical framework explicit. The integrative theory of mismatch captures how organisms track environments across space and time on multiple scales in order to maintain an adaptive match to the environment, and how failures of adaptive tracking lead to disease. In this review, we try to present this complex body of theory as clearly and simply as possible with the aim of facilitating its application in new domains. We introduce terminology, which is as far as possible consistent with earlier usage, to distinguish the different forms of mismatch. Mismatch in its modern form is a productive organizing concept that can help researchers articulate how physiology, development and evolution interact with one another and with environmental change to explain health outcomes.

## 1. THE DUAL ORIGINS OF MISMATCH

The capacity of organisms to adapt to their environments is sometimes outpaced by the speed and scale of environmental change. The likelihood of ‘adaptive lag’, in which evolution by natural selection fails to keep pace with a changing selective environment, was evident to the founders of the Darwinian ‘modern synthesis’ [1]. Adaptive lag provided these authors with a plausible explanation of many cases of observed maladaptation.

The idea of adaptive lag was prominent in thinking about human health well before the emergence of evolutionary medicine as it is now understood [2, 3]. In the 1950s, human geneticist Neel initiated the study of ‘[t]he genetic significance of changing dietary patterns’. Neel’s primary motivation was the opportunity these patterns created to study natural selection in the human species [4, see esp. pp. 43–44]. But he was aware of the medical implications when he argued that high levels of dietary fat and salt in modern environments impose novel selection pressures on human metabolism, leading to reduced reproductive fitness. He suggested that European populations may have partially adapted to these novel diets and that the rapid modernization in other parts of the world might have more severe health consequences [4]. In the 1960s, the psychologist Bowlby introduced the term ‘environment of evolutionary adaptedness’ and argued that, ‘[w]e can … be fairly sure that none of the environments in which civilised, or even half-civilised, man lives today conforms to the environment in which man’s environmentally stable behavioural systems were evolved and to which they are intrinsically adapted’ [5].

The term ‘mismatch’, however, was only first used as an alternative to ‘adaptive lag’ in 1988 [6]. It was used in this sense in Williams and Nesse’s influential 1991 paper ‘The Dawn of Darwinian Medicine’ [2] and their still more influential 1994 book Why We Get Sick [3]. The two terms are now used interchangeably: ‘it is a truism that all organisms must experience some adaptive lag, here meaning a mismatch between current selection pressures and behavior’ [7]. Following Riggs [8], we refer to this mismatch between genes and environment due to adaptive lag as an evolutionary mismatch.

A decade after the term ‘mismatch’ was adopted in evolutionary medicine, a distinction began to be drawn between this evolutionary mismatch between genes and environment and the ‘mismatch between body and environment’ [9] that results from failures of phenotypic plasticity. This idea emerged from work on the Developmental Origins of Health and Disease (DOHaD), an area of biomedical research that studies how events early in the lifecourse influence later health outcomes [10]. Following Kuzawa [11], we refer to this idea as developmental mismatch [see also [12]].

Type II diabetes was a prominent example in these early discussions of mismatch phenomena. Neel’s ‘thrifty genotype’ hypothesis [13] attributed rises in the incidence of Type II diabetes to a mismatch between the modern nutritional environment and genes adapted to an ancestral nutritional environment. The thrifty genotype was an evolutionary mismatch hypothesis, although the term ‘mismatch’ was not used to describe it for another twenty-four years. The later ‘thrifty phenotype’ hypothesis [14] attributed spikes in the incidence of Type II diabetes to the existence of birth cohorts with ill-nourished mothers but lives of nutritional abundance. Barker and collaborators hypothesized that the developing foetus responds to maternal cues of a poor nutritional environment by developing a physiology that is well adapted to survival in those conditions. If, however, the foetus grows up to experience nutritional abundance, these physiological settings increase the risk of Type II diabetes, obesity, and other aspects of ‘metabolic syndrome’.

The thrifty phenotype hypothesis was first described as a ‘mismatch’ between the physiological settings adopted by the foetus and the nutritional environment of the adult in 2000 by Bateson, a distinguished ethologist with a strong interest in behavioural development [9, 15]. Bateson was interested in applying his ideas about the interaction of development and evolution to the new field of Evolutionary Medicine [16]. The term ‘mismatch’ was applied again to DOHaD phenomena in a 2004 manifesto published in Nature [17]. This manifesto resulted from a workshop organized by Bateson with behavioural ecologists, evolutionary biologists (particularly experts on the evolution of phenotypic plasticity), and leading DOHaD researchers, including Barker, originator of the thrifty phenotype hypothesis, and Gluckman, one of its most influential advocates. Afterwards, the term spread rapidly via high-profile publications in the DOHaD literature [18–21] and books explaining the DOHaD paradigm to a wider audience [22, 23].

The existence of two different ‘mismatch’ hypotheses to explain Type II diabetes necessitated terminology to mark the difference between them. Bateson contrasted mismatch between gene and environment (our evolutionary mismatch) with ‘mismatch between body and environment’ [9]. But Kuzawa [11] called this second kind of mismatch ‘developmental mismatch’, as did Gluckman *et al.* in Principles of Evolutionary Medicine [12]. We follow this usage in the remainder of the paper.

**Figure 1.**
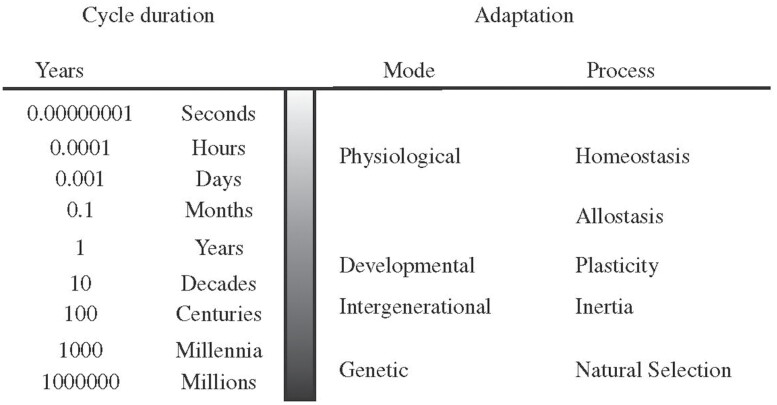
Kuzawa’s representation of adaptation on multiple timescales. The left side shows timescales on which organisms experience ‘ecological change’. The right side shows the corresponding ‘mode of adaptation’ by which organisms can track change on these timescales. ‘Inertia’ is Kuzawa’s term for epigenetic inheritance across two or more generations (Reproduced from Ref. [11]).

But evolution and development are only two of the possible timescales on which a phenotype can be mismatched to an environment. Kuzawa proposed a continuum of timescales (Fig. 1) and a series of mechanisms that produce, or fail to produce, an adaptive match between phenotype and environment on different timescales. Adaptive evolution by natural selection is the slowest of these processes, and homeostatic adaptation is the fastest. The failure of any of these mechanisms can produce a mismatch between a phenotype and the environment.

**Figure 2.**
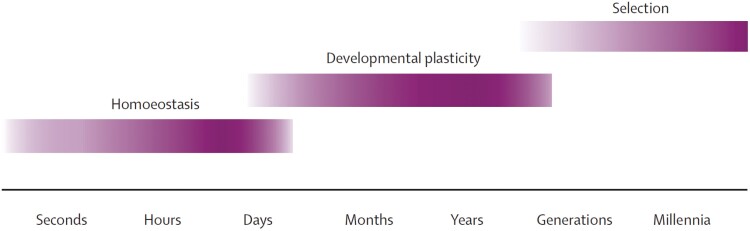
‘Modes of Adaptation’ (Reproduced from Ref. [24]).

Other theorists reached the same conclusion. Following the success of the 2004 Nature paper, Gluckman convened a larger and overlapping gathering of ‘clinicians and public-health specialists from high-income and low-income countries, developmental and evolutionary biologists, geneticists, anthropologists, and economists’ [24]. The resultant twenty-authored manifesto ‘Towards a new developmental synthesis: Adaptive developmental plasticity and human disease’ was published in the Lancet. It endorsed the idea that organisms use multiple ‘modes of adaptation’ to track environments that change simultaneously on multiple timescales (Fig. 2).

The key development in this work was the realization that organisms can adapt to an environmental challenge either by evolution or by physiological or developmental adaptation and that these modes of adaptation interact. This idea is now commonplace in evolutionary medicine. Research on nutrition notes that ‘[m]ismatch occurs when the timescale and/or magnitude of environmental change exceeds the combined capacity of adaptation owing to homeostatic mechanisms, phenotypic plasticity and transgenerational adaptation’ [25]. Research on populations at high altitude notes that ‘[k]nowing how long the population has resided at altitude is important for considering the potential for modes of adaptation that occur on time scales ranging from short (reversible acclimatization), to intermediate (developmental) and long (genetic)’ [26]. While introductory presentations of Evolutionary Medicine still emphasize the evolutionary timescale, they acknowledge that mismatch can also be identified on developmental timescales: ‘evolutionary mismatch, [is] defined here as the phenomenon by which previously adaptive alleles are no longer favoured in a new environment … other uses of mismatch are applied over the life course’ [27].

In contemporary evolutionary medicine, we can see this sophisticated theory of mismatch on multiple timescales at work. Just as Bateson hoped, it allows insights to flow in both directions between evolutionary theory and the study of development and physiology. Two examples of this mutual illumination are, first, that the goals built into mechanisms of homeostasis and allostasis are trade-offs between multiple life-history goals and, second, that mechanisms of physiological and developmental adaptability shape the selection pressures acting on genotypes (see Section 4).

The concept of mismatch contributes to the defining project of evolutionary medicine, which is to analyse and explain susceptibility to disease [3]. One obvious cause of susceptibility to disease is maladaptation to the environment—an organism that loses an arms race with its parasites, or which is unable to obtain the foodstuffs that its digestive system evolved to process, is likely to suffer from pathology. It is for this reason that ‘Mismatch’ occurs in the lists of ‘pathways to disease’ that structure evolutionary medicine textbooks [e.g. [12] (2nd ed. 2016), [28]]. Mismatch is a way to explain the existence of maladaptive phenotypes within a broad adaptationist [29] framework which expects that, as a result of natural selection, both constitutive phenotypes and mechanisms of plasticity will be well adapted to their environments. So mismatch is one important way to explain susceptibility to disease in evolutionary medicine.

## 2. REFERENCE ENVIRONMENTS FOR MISMATCH

**Figure 3.**
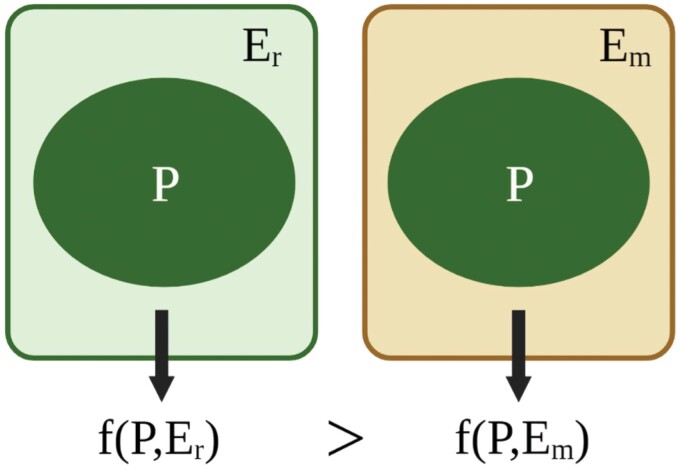
Biological applications of the term ‘mismatch’ conform to a general schema: a biological unit with phenotype P is in a state of mismatch with respect to its actual environment *E*_*m*_ for a given utility function f if, considering a reference environment *E*_*r*_, *P* performs worse with respect to *f* in *E*_*m*_, than it would in the reference environment.

In its most abstract form mismatch requires a phenotype in an environment and another reference environment in which the phenotype does better than in its actual environment. It also requires a currency, which is usually biological fitness (see Fig. 3).

When evolutionary medicine studies change on evolutionary timescales, the reference environment for the mismatch is the historical environment to which the organism is adapted, often referred to as the ‘environment of evolutionary adaptedness’ (EEA). The EEA concept has been criticized because a species can occupy many different environments in the period over which a trait evolves and also because of niche construction [7, 30, 31]. The primary target of these criticisms is the use of the EEA concept in evolutionary psychology, although naive understandings of the EEA also underlie a number of fallacies about human health [32]. According to Buller, evolutionary psychologists regard the EEA as a specific set of environmental factors, and this way of thinking can also be seen in the first paper to use the term ‘mismatch’ in evolutionary medicine [6]. However, the EEA is not usually conceived as a specific set of environmental factors, let alone a specific place and time in history, but rather as a set of parameters in a model of natural selection. The values of those parameters represent a weighted sum of the environments experienced by the organism over the period of time relevant to explaining the currently observed value of the phenotype. For example, in studying the evolution of adaptations to high altitude, researchers seek to establish historical occupation patterns across the diverse landscapes that have been inhabited by populations that exhibit these adaptations [26]. The EEA of a population is a set of parameters that summarize the available information about these patterns of occupation. This example also shows that spatial change in the environment—for example migration—must be taken into account when thinking about mismatch as well as temporal change. The ability to determine the parameters of the EEA is an important constraint on the rigorous use of evolutionary mismatch explanations. One relatively unproblematic application of the concept is when parameters that have been more or less constant through human evolution have changed due to documented recent developments, such as the virtual elimination of multicellular gut parasites in the developed world.

But when evolutionary medicine studies change on developmental timescales, the reference environment need not be the EEA. In developmental mismatch, a phenotypically plastic organism develops in a way that does not match its environment, resulting in a maladaptive phenotype. Consider a water flea that grows defensive armour in response to chemical cues of the presence of predators, and then finds itself in a pool with no predators. The flea would have been better off had it saved these resources and invested them in reproduction. There are in principle two ways in which adaptive developmental plasticity can produce a phenotype that does not match its environment. One is because the organism manifesting the plastic response is not in its EEA, something that may lead all or most individuals to develop the ‘wrong’ phenotype. For example, a change in the environment can disrupt the relationship between cues earlier in life and environments later in life. The organism that relies on that cue is evolutionarily mismatched to the new environment. This will lead to many individuals in the population being developmentally mismatched to their local environment. In such cases, organisms are mismatched both at the population and individual levels.

The second way in which adaptive developmental plasticity can produce a phenotype that does not match its environment is when the EEA is ‘normal but noisy’ (as in the water flea example). Most developmental decisions must be made with less than-perfect information, so even an organism following an optimal decision rule will make errors at a predictable rate. Matthewson and Griffiths [33] have argued that the inevitability of errors in mechanisms of ‘predictive adaptive responses’ (PAR) is a major original insight of evolutionary medicine. Organisms will sometimes be mismatched even in their EEA because phenotypic plasticity uses (noisy) cues to predict which of a range of (normal) environments the organism will encounter in later life and these predictions are less than perfect [18].

The concept of PAR, in which an organism develops in response to cues that predict future environmental conditions, has been very prominent in discussions of developmental mismatch. However, Bateson and Nettle [34] have pointed out that there are other important cases of adaptive developmental plasticity that can give rise to developmental mismatch. They distinguish between informational adaptive developmental plasticity, a category that includes but is not exhausted by PARs, and somatic state-based adaptive developmental plasticity. The latter does not involve a prediction about the future state of the environment, but only the recognition that the organism’s current somatic state will persist. For example, if an individual is small relative to its conspecifics and will stay small throughout its reproductive career, then it will pay to adopt a life-history strategy that takes its small size into account. Bateson and Nettle’s distinction has important implications for formulating and testing evolutionary hypotheses about adaptive developmental plasticity. For present purposes, however, we simply note that somatic state-based adaptive developmental plasticity can give rise to developmental mismatch for both the reasons we have described. First, a response to a somatic state that maximized fitness in the EEA may no longer do so in a new environment. Second, an organism may make an error about its own somatic state. It is surely possible that an environmental change could disrupt the relationship between a cue of a somatic state and an actual somatic state, although it is much easier to see how this will happen in informational adaptive developmental plasticity.

**Figure 4.**
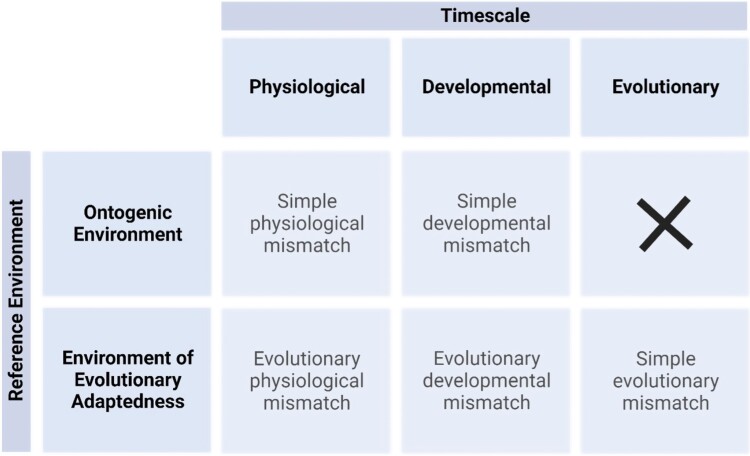
A taxonomy of mismatch phenomena. See text for explanation and examples.

While the earlier literature did not have distinct names for the two different scenarios that can account for an observed developmental mismatch, the two alternative scenarios were clearly distinguished and the point that even under ideal circumstances development involves committing to a phenotype with less than-perfect information was clearly recognized [e.g. 18, 11]. In the next section, we introduce some terminology to distinguish these different mismatch scenarios (Fig. 4 and Table 1).

**Table 1.** Glossary

‘Developmental Origins of Health and Disease’ (DOHaD)	Biomedical research paradigm that studies how events early in the lifecourse influence later health outcomes.
Constitutive phenotype	Phenotype that is expressed in all normal developmental environments.
Environment of Evolutionary Adaptedness (EEA)	A set of parameters in a model of natural selection that represents the weighted set of environments to which a phenotype is adapted.
Ontogenetic environment	The parameters in a model of individual development that interact with genotype to determine phenotype. In a patchy environment, different individuals experience different ontogenetic environments. Some of these may be ‘hostile’ environments, in the sense that they reduce the fitness of the individual relative to individuals who develop in ‘benign’ environments.
Adaptive lag	Occurs when the environment changes more rapidly than a population can adapt by natural selection. Adaptive lag can explain why a population is maladapted in its current environment.
Expected fitness	For our purposes, the average lifetime reproductive output of an organism with a particular genotype or phenotype, assuming a ‘patchy’ environment: that is, one containing multiple states. More complex metrics of fitness exist.
Realized fitness	The actual lifetime number of offspring produced by an organism. If applied to more than one individual, it assumes that all these organisms have been subjected to the same environmental conditions in an otherwise patchy environment. If the environment has a single state (and excluding genetic drift) expected and realized fitness are identical.
Phenotypic plasticity	A phenotype whose expression differs in response to the particular environmental conditions to which an organism is exposed. Plasticity may occur on both developmental and physiological timescales.
Mismatch	Situation in which fitness is decreased as a result of an environmental change (temporal or spatial) that occurs more rapidly than a population can adapt.
Evolutionary mismatch	**Mismatch** in which the utility function is expected fitness and the reference environment is the Environment of Evolutionary Adaptedness (EEA). Evolutionary mismatch occurs whenever Adaptive Lag leads to a reduction of fitness.
Simple evolutionary mismatch	**Evolutionary mismatch** occurring with a constitutive phenotype.
Evolutionary developmental mismatch	A form of **evolutionary mismatch** that has attracted particular attention in evolutionary medicine. Environmental change disrupts the relationship between cue and consequence implicitly assumed by a mechanism of developmental plasticity, leading that mechanism to consistently generate maladapted phenotypes.
Simple developmental mismatch	**Mismatch** in which the utility function is realized fitness and the mechanism responsible for the mismatch is phenotypic plasticity. Simple Developmental Mismatch will occur regularly in the EEA because developmental decisions are made with less than perfect information.
Evolutionary physiological mismatch	The phenomenon, included in the definitions of mismatch by Kuzawa [11], Gluckman *et al.* [12] and Raubenheimer, Simpson and Tait [25], in which environmental change has disrupted the relationship between cue and consequence assumed by physiological mechanisms of homeostasis or allostasis so that these mechanisms consistently generate maladaptive phenotypic changes.
Simple physiological mismatch	For completeness, we note that failures of homeostatic or allostatic mechanisms in the EEA could logically be described as ‘simple physiological mismatch’.

## 3. A TAXONOMY OF MISMATCH PHENOMENA

We call a mismatch that occurs on the evolutionary timescale and to a constitutive phenotype a ‘simple evolutionary mismatch’ (Fig. 4 and Explanatory Box). The reference environment here is the EEA. Organisms living outside the parameters of the EEA will often have reduced fitness, which will often manifest as poor health outcomes. The thrifty genotype hypothesis is an example of a simple evolutionary mismatch.

When a mismatch occurs on the developmental timescale because natural selection has failed to keep pace with the changing environment we call this an ‘evolutionary developmental mismatch’ (Fig. 4 and Explanatory Box). Once again, the reference environment is the EEA. Natural selection has not adapted the mechanisms underlying developmental plasticity to the changing environment. This failure of adaptation on an evolutionary timescale explains population-wide failures of adaptation on the developmental timescale.

The thrifty phenotype hypothesis is usually presented as an example of evolutionary developmental mismatch, with modern environments systematically giving the developing foetus the wrong cue about its future environment. But researchers have emphasized that even in normal environments this mechanism may sometimes produce a thrifty phenotype that finds itself living in nutritional abundance [18]. This case would be an example of our next category of mismatch.

When a mismatch occurs on the developmental timescale simply because a mechanism of phenotypic plasticity has produced the wrong phenotype for the actual environment we call this a ‘simple developmental mismatch’ (Fig. 4). Such failures occur because even a perfectly adapted mechanism for making decisions under uncertainty must make some errors [33]. No evolutionary mismatch is needed. Both matched and mismatched organisms are ex hypothesi in the EEA. The difference between them is that one successfully matches its phenotype to the patch in which it finds itself, while the other does not. Hence, in this case, the actual environment (*E*_*m*_, see Fig. 3) is the environment of the patch in which the organism develops and does badly whilst the reference environment (*E*_R_) is the environment of a patch in which an organism with that phenotype would thrive.

The distinction between evolutionary developmental mismatch and simple developmental mismatch can also be understood in terms of which measure of fitness is used. In evolutionary developmental mismatch, we compare the success of the same phenotype in current and ancestral environments, which is equivalent to comparing the expected fitness of the phenotype in these two environments. In simple developmental mismatch, we compare the success of two individuals with the same phenotype but different individual environments, which is equivalent to comparing the realized fitness of two individuals (see Ref. [35]).

We can make the same distinction between simple and evolutionary mismatch for mismatch phenomena occurring on the physiological timescales (Figs. 2 and 4) and we discuss some examples of each in Section 4.

It is important to note that while we have distinguished three timescales—physiological, developmental and evolutionary—this division is not rigid. Kuzawa [11] adds a fourth, ‘intergenerational’ timescale (Fig. 1) for mismatches generated by mechanisms of inter-generational adaptive plasticity (‘parental effects’ [36]). In contrast, Raubenheimer, Simpson and Tait [25] treat epigenetic and genetic mechanisms as operating on a single ‘transgenerational’ timescale. The important point is that mismatch can occur as a result of many different mechanisms, each of which acts on a specific timescale. Finer or coarser-grained timescales can be used to study particular aspects of mismatch.

## 4. HOW MISMATCH INTEGRATES DEVELOPMENT AND EVOLUTION

Tinbergen’s ‘four questions’ framework for biological research [37] has been an important inspiration for evolutionary medicine [38]. Answers to the four questions (Mechanism, Development, Survival Value, Evolution) should be mutually illuminating. One of the strengths of an integrative theory of mismatch on multiple timescales is that it highlights numerous ways in which evolution, development and physiology interact to explain health and disease.

We have already encountered one way in which mismatch connects evolution and development: evolutionary mismatch can cause developmental mismatch. In evolutionary developmental mismatch, a mechanism of developmental plasticity produces a mismatched phenotype because the cue-consequence relationship implicitly assumed by the mechanism does not hold in the new environment. For example, the mechanisms by which infants use cues in milk to form taste preferences are mismatched with the environment of formula feeding. Avoiding tastes not found in infant formula, such as the tastes of vegetables [39], is maladaptive. Evolutionary developmental mismatch can have very dramatic consequences: apparently insignificant changes to the environment may have dramatic consequences for population health if organisms use them as cues to determine their developmental trajectory. This insight is at the heart of DOHaD research on PAR [20, 40].

But developmental mismatch is not just a special case of evolutionary mismatch. It is an independently defined phenomenon that only sometimes results from evolutionary mismatch. Simple evolutionary mismatch has potentially very different implications for medicine. As we explained in Section 2, developmental decisions are almost always decisions under uncertainty so that even an optimal decision rule generates errors. Gluckman and collaborators argued that a predictive adaptive response may produce ‘a continuous range of human metabolic ““morphs”” representing a suite of integrated responses to the environmental cues received in utero or by the neonate which establish the setpoints of the metabolic and related systems’ [40]. As a result, ‘even when fetal growth falls within the normal range, being born into an enriched postnatal environment can create a mismatch’ [18]. So a PAR will make errors, not because it is operating in a novel environment, but because the best possible decision rule still has a rate of error. These errors are simple developmental mismatches.

Mismatches can also occur on physiological timescales. For example, an organism that has elevated cortisol levels in response to cues of a hostile environment but which is actually living in a benign environment is mismatched to that environment. This organism will pay the short-term physiological cost of increasing cortisol level on realized fitness when this is unnecessary.

The theory of mismatch draws attention to the parallels between such physiological mismatches and mismatches on larger timescales. Suppose that some failure of homeostasis or allostasis occurs strikingly often in a population, and hence calls for an explanation. One possible explanation is a downstream consequence of evolutionary mismatch or ‘evolutionary physiological mismatch’. This possibility would involve an evolutionary mismatch between the physiological mechanism and a novel environment because the cue-consequence relationship implicit in the design of a physiological mechanism no longer holds in that environment. The other possible explanation parallels ‘simple developmental mismatch’. In ‘simple physiological mismatch’, a physiological mechanism implements a rule that maximizes expected fitness but because the environment is noisy even the optimal rule often fails to maximize realized fitness (see Fig. 4). The important ‘smoke detector principle’ in evolutionary medicine is one example of how a mismatch in physiological timescales is the inevitable result of ‘normal but noisy’ environments [41].

The theory of mismatch integrates all these phenomena into a theory of how physiology, development and evolution interact with each other and with environmental change to explain health outcomes. As an example of the power of this integrative approach, consider how it improves our understanding of homeostasis. Once we see homeostasis as a mechanism of fitness-tracking on short timescales we can analyse it in the framework of life-history theory, the same framework we use to understand the evolution of constitutive phenotypes and of developmental plasticity. It is ultimately inadequate to regard homeostasis (and its relatives homeorhesis, allostasis, etc) as targeting anything other than the maximization of expected fitness [25]. The values and ranges of internal variables that are the targets for these physiological mechanisms are not optimal in any simple, physiological sense, but rather represent trade-offs between multiple goals where the implicit relative fitness payoffs of those goals are those from the EEA.

A powerful example of this evolutionary perspective on physiological mechanisms comes from nutrition science. From an evolutionary perspective the idea that nutrition science should identify an ‘optimal diet’ appears naïve. It is a fundamental mathematical fact about life-history theory that a number of distinct functions are being optimized simultaneously. At the most basic level, lifespan and reproductive output cannot both be maximized and the optimal life-history strategy represents a trade-off between these two functions. In the case of nutrition, different macronutrient ratios are ‘optimal’ for maximum lifespan, for maximum lifetime reproductive output, and for high immune functioning [42]. The observed nutritional intake target, which is itself a complex plastic trait that changes over the lifecourse, will represent a trade-off between these different goals that reflects their relative importance in the EEA.

As well as integrating evolutionary understanding into the study of development and physiology the theory of mismatch integrates physiological and developmental understanding into the study of evolution. An organism with a mechanism of physiological or developmental plasticity that allows it to adapt to some range of environmental change does not need to undergo adaptation by natural selection in that range of environments. Thus, in order to predict the response to selection biologists need to take into account the capacity of an organism for physiological and developmental adaptation. Once again, this idea finds powerful applications in nutrition science [25].

Physiological plasticity, developmental plasticity, and evolution by natural selection are all means to the same end—tracking changing environments so as to maximize fitness. The design of each of these mechanisms reflects the same evolutionary compromises between multiple life-history goals. These mechanisms interact with one another, forming a suite of means by which organisms can cope with environmental heterogeneity. It is this focus on adaptive tracking, and on multiple mechanisms and modes of adaptation, that we take to be the distinctive core of research into mismatch in evolutionary medicine.

## 5. CONCLUSION

The theory of mismatch found in contemporary evolutionary medicine merges two ideas that were both labelled with the term ‘mismatch’ sometime after they were recognized and discussed in the scientific literature. The first idea is ‘evolutionary mismatch’ [8]: adaptation by natural selection sometimes fails to keep pace with environmental change. This idea emerged in the modern synthesis, where it was known as ‘adaptive lag’. The second idea is ‘developmental mismatch’ [11]: mechanisms of developmental and phenotypic plasticity produce maladaptive phenotypes when a cue early in development does not correspond to the actual circumstance later in the lifecourse. These developmental mismatches are sometimes (but not always) the result of an evolutionary mismatch between the mechanism of plasticity and the modern environment. This second idea originated in the field of DOHaD. The first idea was first labelled ‘mismatch’ in the late ‘80s, and the second was first labelled ‘mismatch’ in the late ‘90s. In the 2000s there was a deliberate effort to synthesize these two research traditions, with interdisciplinary workshops and multi-author ‘manifesto’ articles in prestigious journals.

In contemporary evolutionary medicine, the idea of mismatch frames the study of how organisms track changing environments on multiple scales so as to maximize fitness, and of where this goes wrong. The resultant body of theory recognizes that evolutionary and developmental and physiological mismatch interacts in numerous ways, and hence that the study of mismatch must integrate evolutionary and developmental and physiological studies. Mismatch is a rich example of how evolutionary, and evolutionary developmental, reasoning can contribute to biological and medical science.
